# Computational modeling of the development of detailed facial representations along ventral pathway

**DOI:** 10.1186/1471-2202-15-S1-P38

**Published:** 2014-07-21

**Authors:** Akihiro Eguchi, Simon M Stringer

**Affiliations:** 1 Oxford Centre for theoretical neuroscience and artificial intelligence, University of Oxford, OX1 3UD, UK

## 

Experimental studies have shown that neurons at an intermediate stage of the primate ventral visual pathway encode the conformation and spatial relations of facial features [1], while neurons in the later stages are selective to the full face [2]. In this study, we investigate how these cell firing properties may develop through visually-guided learning.

**Figure 1:**
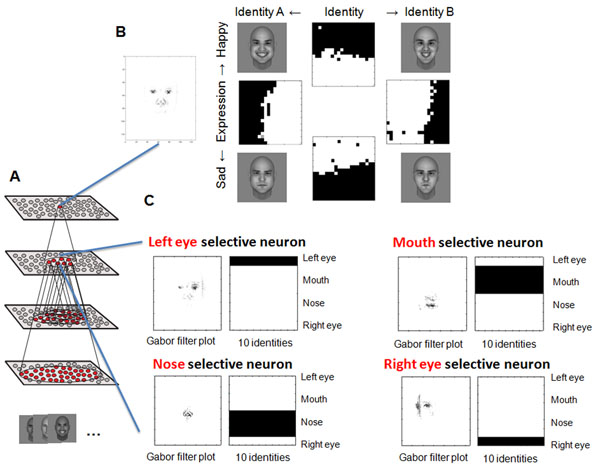
**A.** Stylized image of the 4 layer network model. **B.** Gabor filter inputs which had strong connectivity through the layers to full face selective neuron (left) Firing activity plots of cells, of which each encodes specific facial identity or expression, across 20 x 20 morphs of distinct identities and expressions. **C.** Gabor filters inputs of facial feature selective neurons and firing activity plots of the neurons across a series of presentations of different facial features within 10 distinct faces.

A hierarchical neural network model of the primate’s ventral visual pathway is trained by presenting many randomly generated faces to the hierarchical competitive neural network while a local learning rule modifies the strengths of the synaptic connections between successive layers [3] (Figure 1A). After training, the model is found to have developed the experimentally observed cell firing properties. In particular, we have demonstrated how the primate brain learns to represent facial expression independently of facial identity as reported in [4] (Figure 1B). We have also shown how the visual system forms separate representations of facial features such as the eyes, nose and mouth (Figure 1C) as well as representations of spatial relationships between these facial features, as have been reported in single unit recording studies [1]. Therefore, this research makes an important contribution to understanding visual processing in the primate brain.
